# The Perils of Methanol Exposure: Insights into Toxicity and Clinical Management

**DOI:** 10.3390/toxics12120924

**Published:** 2024-12-20

**Authors:** Mohammed Alrashed, Norah S. Aldeghaither, Shatha Y. Almutairi, Meshari Almutairi, Abdulrhman Alghamdi, Tariq Alqahtani, Ghada H. Almojathel, Nada A. Alnassar, Sultan M. Alghadeer, Abdulmajeed Alshehri, Mohammed Alnuhait, Omar A. Almohammed

**Affiliations:** 1 Department of Pharmacy Practice, College of Pharmacy, King Saud bin Abdulaziz University for Health Sciences, Riyadh 14611, Saudi Arabia; aldeghaither263@ksau-hs.edu.sa (N.S.A.); almoteri040@ksau-hs.edu.sa (S.Y.A.); almutairi0217@ksau-hs.edu.sa (M.A.); shehriabdul@ksau-hs.edu.sa (A.A.); 2 Pharmaceutical Care Department, King Abdulaziz Medical City, National Guard Health Affairs, Riyadh 11426, Saudi Arabia; 3 King Abdullah International Medical Research Center, Riyadh 11481, Saudi Arabia; qahtanita@ksau-hs.edu.sa; 4 Emergency Medical Services Department, College of Applied Medical Sciences, King Saud bin Abdulaziz University for Health Sciences, Riyadh 14611, Saudi Arabia; ghamdia@ksau-hs.edu.sa; 5 Department of Pharmaceutical Sciences, College of Pharmacy, King Saud bin Abdulaziz University for Health Sciences, Riyadh 14611, Saudi Arabia; 6 Department of Clinical Pharmacy, College of Pharmacy, King Saud University, Riyadh 12371, Saudi Arabia; 439200038@student.ksu.edu.sa (G.H.A.); 439200129@student.ksu.edu.sa (N.A.A.); salghadeer@ksu.edu.sa (S.M.A.); oalmohammed@ksu.edu.sa (O.A.A.); 7 Pharmaceutical Practices Department, College of Pharmacy, Umm Al-Qura University, Makkah 21955, Saudi Arabia; manuhait@uqu.edu.sa; 8 Pharmacoeconomics Research Unit, College of Pharmacy, King Saud University, Riyadh 12371, Saudi Arabia

**Keywords:** methanol, toxicity, pathophysiology, clinical presentation, diagnostics, management

## Abstract

Methanol is a widely used industrial and household alcohol that poses significant health risks upon exposure. Despite its extensive use, methanol poisoning remains a critical public health concern globally, often resulting from accidental or intentional ingestion and outbreaks linked to contaminated beverages. Methanol toxicity stems from its metabolic conversion to formaldehyde and formic acid, leading to severe metabolic acidosis and multiorgan damage, including profound CNS effects and visual impairments. Epidemiological data underscore the widespread impact of methanol poisoning, with alarming case fatality rates reported in various countries. Comprehensive prevention and effective management strategies are urgently needed to address the significant morbidity and mortality associated with methanol poisoning. The clinical manifestations of methanol toxicity vary between adult and pediatric populations and between acute and chronic exposure. Adults typically present with gastrointestinal and neurological symptoms, whereas pediatric patients often exhibit more severe outcomes due to differences in metabolism and body weight. The diagnosis of methanol poisoning involves a combination of clinical evaluation, laboratory testing, and advanced diagnostic techniques. The identification of metabolic acidosis, elevated anion and osmolal gaps, and confirmation through methanol and formate levels are critical for accurate diagnosis. Timely intervention is crucial, and the management of methanol poisoning includes securing the airway, breathing, and circulation; addressing metabolic acidosis with sodium bicarbonate; administering antidotes such as fomepizole or ethanol; and administering hemodialysis, which plays a pivotal role in eliminating methanol and its toxic metabolites, especially in severe cases. The complexity of methanol poisoning necessitates a comprehensive approach encompassing early recognition, prompt intervention, and coordinated care among healthcare providers. Increased awareness, effective prevention strategies, and timely treatment protocols are essential to mitigate severe health consequences and improve patient survival and recovery.

## 1. Introduction

Methanol is a toxic alcohol found in various products. Methanol poisoning occurs through accidental or intentional ingestion, leading to outbreaks. This global health concern contributes to significant morbidity and mortality. Common products containing methanol include windshield washer fluid, gas line antifreeze, carburetor cleaner, copy machine fluids, perfumes, food warming fuels, and other types of fuel. A variety of detrimental consequences are associated with methanol poisoning, necessitating measures such laboratory monitoring, antidotal therapy, and emergency dialysis. The severity of methanol poisoning highlights the urgent need for comprehensive preventive measures and effective management strategies to address its consequences, including permanent neurological damage, blindness, and even death [1].

The global consumption of methanol exceeds 225 million liters per day, and in recent years, methanol poisoning outbreaks in many developed countries have increased alarmingly [2]. Methanol poisoning presents differently in adult and pediatric populations and varies between acute and chronic exposure. Although uncommon among pediatric patients, methanol toxicity has been reported with varying severity. In mild cases, there is a minimal decrease in central nervous system (CNS) activity, accompanied by weakness, dizziness, and nausea. Severe cases lead to metabolic acidosis, which manifests as blurred vision, bilateral mydriasis, gastrointestinal (GI) symptoms, disorientation, visual abnormalities, photophobia, and sporadic blindness [3]. Adults may present symptoms such as nausea, vomiting, abdominal pain, and neurological disturbances, whereas pediatric patients pose unique challenges due to differences in metabolism and body weight, potentially resulting in more severe manifestations and poorer outcomes [4]. Acute poisoning typically involves a single large ingestion, resulting in rapid onset of symptoms such as metabolic acidosis, visual disturbance, and CNS depression. Conversely, chronic poisoning results from repeated low-level exposures over time, leading to insidious symptoms such as chronic headaches, dizziness, and progressive visual impairment [5].

This review aims to examine methanol toxicity, focusing on the proper evaluation of methanol poisoning cases, the contributing factors and circumstances leading to exposure, the effectiveness of current treatment protocols, challenges in diagnosis and management, and preventive strategies to reduce methanol-related incidents in various regions. Understanding these aspects is crucial for improving outcomes and implementing effective public health measures.

## 2. Pathophysiology

**Figure 1:**
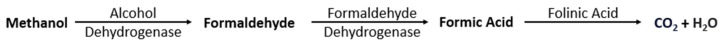
The metabolism of methanol in the liver.

Methanol poisoning manifests as a complex pathophysiological sequence. Methanol is rapidly absorbed through the GI tract, causing nausea, vomiting, and abdominal pain, and subsequently enters the CNS, causing confusion and drowsiness. Methanol undergoes metabolism through several enzymatic processes in the liver. This series of oxidation reactions results in the accumulation of more toxic metabolites (Figure 1) [1].

The key driver of the pathophysiological effects of methanol poisoning is the accumulation of formic acid, which leads to metabolic acidosis. This condition interferes with normal cellular metabolism and can result in harmful consequences for various organ systems. The CNS is particularly vulnerable to the consequences of methanol poisoning. Formic acid and formaldehyde can lead to neurological symptoms, including headache, confusion, seizures, and, in severe cases, coma. These CNS effects contribute to direct damage to brain tissues and neurotransmitter imbalances. Another hallmark of methanol poisoning pathophysiology is visual impairment, including blindness. If not promptly addressed, this damage can become irreversible [1,6].

Methanol poisoning is not limited to the CNS; it can impact multiple other organ systems. The cardiovascular system may experience hemodynamic instability, and acute kidney injury may occur because of the systemic influence of acidosis and the accumulation of toxic metabolites. Timely intervention is crucial when managing methanol poisoning cases. Antidotes such as ethanol or fomepizole are administered to inhibit the activity of alcohol dehydrogenase (ADH), effectively halting the further breakdown of methanol. This intervention reduces the formation of toxic metabolites and mitigates acidosis, CNS effects, and multiorgan dysfunction [4,6]. Ethanol administration plays a crucial role in inhibiting methanol metabolism due to its competitive binding with ADH. Ethanol has a higher affinity (lower Km) for ADH compared to methanol, effectively outcompeting methanol for enzymatic binding. This prevents the oxidation of methanol into its toxic metabolites, such as formaldehyde and formic acid, thereby reducing their harmful effects. Fomepizole, on the other hand, is a direct inhibitor of ADH and works by completely halting the enzymatic activity of ADH. Both interventions are essential for mitigating metabolic acidosis, CNS effects, and multiorgan dysfunction associated with methanol poisoning [4,6].

## 3. Toxicokinetic

Methanol toxicity primarily occurs through ingestion, although exposure can also happen via inhalation or skin absorption. For an adult, a potentially fatal dose is approximately 1 g per kilogram of body weight [1,7]. Once ingested, methanol is rapidly absorbed from the GI tract, reaching peak concentrations within 30 to 90 min. Due to its small molecular size and lipophilic nature, methanol readily crosses the blood–brain barrier, leading to severe CNS effects in poisoning cases [8].

The liver plays a critical role in methanol pharmacokinetics as the primary site for its metabolism. Hepatic alcohol dehydrogenase (ADH) oxidizes methanol into formaldehyde, a highly toxic intermediate metabolite [9]. Formaldehyde is then rapidly oxidized into formic acid [10]. At physiological pH, formic acid dissociates into formate and a hydrogen ion. A folate-dependent system further metabolizes formate, ultimately producing water and carbon dioxide (Figure 1). This metabolic pathway begins with the interaction of formate and tetrahydrofolate, leading to the formation of 10-formyl tetrahydrofolate [11].

Formate oxidation depends on hepatic tetrahydrofolate levels, which are regulated by two key factors: the availability of dietary folic acid and the efficiency of tetrahydrofolate regeneration during formate oxidation [12]. The enzyme 10-formyl tetrahydrofolate dehydrogenase plays a pivotal role in recycling tetrahydrofolate and catalyzing the final step of formate oxidation [11]. Studies have shown that folic acid supplementation can reduce the toxic effects of methanol and enhance formate oxidation in various species, including humans and monkeys [13]. These findings highlight the critical role of formic acid in methanol toxicity and suggest that folate supplementation may be beneficial in treating methanol poisoning [14]. Methanol oxidation leads to the formation of formaldehyde, which is subsequently converted into formic acid.

Formic acid, a primary toxic metabolite of methanol, inhibits cytochrome oxidase, a critical enzyme in the electron transport chain responsible for oxidative phosphorylation. This inhibition disrupts mitochondrial energy production, leading to reduced ATP synthesis and cellular energy failure. High-energy-demand organs, such as the brain and retina, are particularly vulnerable to this effect. Furthermore, cytochrome oxidase inhibition contributes to the accumulation of lactate and exacerbates metabolic acidosis, further impairing cellular function. These highly reactive metabolites can also quickly bind to tissue proteins [8]. During methanol metabolism, the osmolar gap decreases while the anion gap increases. The development of anion gap metabolic acidosis due to formic acid accumulation is a complex process. It involves the buildup of organic acids, such as formic acid and formate, which are difficult to eliminate. Moreover, formate disrupts oxidative phosphorylation by inhibiting cytochrome oxidase, thereby impairing mitochondrial respiration. This disruption can lead to elevated lactate levels, which facilitates the passage of formic acid across the blood–brain barrier [1,15,16].

The half-life of methanol is approximately eight minutes, with peak blood levels reached rapidly after ingestion, before declining [17]. Methanol elimination generally follows zero-order kinetics due to the saturation of ADH. Limited data indicate elimination rates ranging from 2.7 mmol/L/h (8.5 mg/dL/h) to 6.3 mmol/L/h (20 mg/dL/h). However, when methanol metabolism is inhibited by antidotes such as ethanol or fomepizole, elimination transitions to first-order kinetics, with a half-life extending from 22 to 87 h. Half-life appears to increase with higher serum methanol concentrations, although the underlying reasons remain unclear [18].

## 4. Clinical Presentation

Like ethanol, methanol acts as a central nervous system depressant. Early symptoms include a general feeling of malaise, accompanied by weakness, headache, and nausea. In some cases, severe symptoms may develop. Most of the methanol in the body is converted into formaldehyde, which is then rapidly metabolized into the highly toxic formic acid and its anion, formate. Extremely high doses can cause severe acidosis, leading to multiorgan failure, brain damage, blindness, and even death [1].

**Figure 2:**
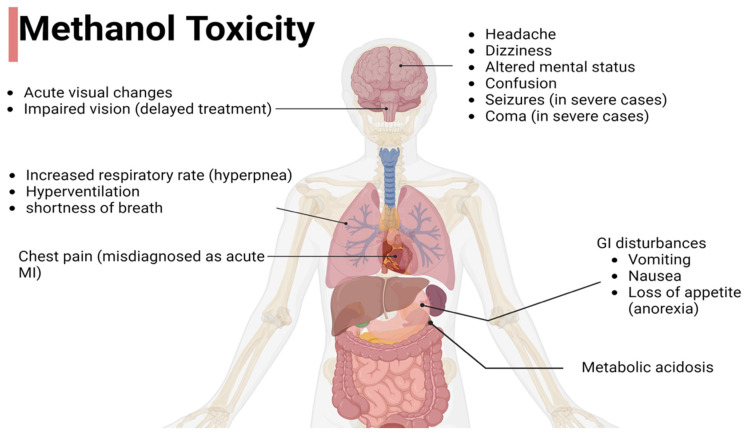
Clinical manifestations of methanol poisoning across various organ systems.

Methanol poisoning can be identified through a range of clinical signs and symptoms, often occurring in distinct phases. In the early stages, which occur within the first few hours following exposure, individuals may experience nonspecific symptoms, such as headache and dizziness, and GI disturbance, such as nausea and vomiting. These early manifestations can be misleading or confusing and may not be immediately recognized as methanol poisoning. However, as toxic metabolites accumulate, more severe and specific symptoms arise. Systemic toxicity includes metabolic acidosis, increased respiratory rate (hyperpnea), loss of appetite (anorexia), headache, and nausea. Hyperventilation may lead to the initial complaint of shortness of breath in many patients. Some individuals may experience chest pain and, as a result, could be initially diagnosed with acute myocardial infarction. The neurological symptoms of these patients often include altered mental status, confusion, and, in severe cases, seizures or coma. Delayed treatment can lead to lasting consequences such as impaired vision and brain damage [1,4,19]. A comprehensive understanding of these clinical signs and symptoms is critical for timely diagnosis and intervention, emphasizing the need for early treatment to mitigate the potentially life-threatening consequences of methanol poisoning (Figure 2).

## 5. Evaluation and Diagnostic Testing

Diagnosing methanol poisoning becomes challenging in the absence of exposure history, particularly when ethanol coingestion occurs, leading to an extended latency period. A comprehensive evaluation approach is needed to assess the severity of methanol toxicity. Clinical evaluation plays a crucial role in the initial assessment, with healthcare professionals considering symptoms such as headache, nausea, vomiting, and visual disturbances. Vital signs are typically normal, and Kussmaul respirations are uncommon even in cases of severe acidosis [20]. Patients who experience severe abdominal pain may exhibit rigidity of the abdominal wall, although rebound tenderness is not usually present [20].

Performing visual acuity and fundoscopy examinations is essential in cases of methanol poisoning. Signs of ocular toxicity include dilated pupils that are partially reactive or nonreactive, as well as optic disc hyperemia with blurred margins resembling pseudopapillitis [20,21]. Microscopic analysis of optic nerves from methanol-poisoned individuals may reveal generalized swelling of intra-axonal mitochondria and clear spaces within the myelin sheath in the retrolaminar portion. These ocular changes are attributed to the direct toxic effects of formate, which inhibits cytochrome oxidase, leading to decreased ATP production and impaired ATP-dependent functions. Inhibition of the sodium–potassium membrane pump disrupts electrical conduction, resulting in early but potentially reversible visual disturbances [22].

A definitive diagnosis of methanol poisoning is made by detecting methanol and formic acid in the blood or urine. A patient who has ingested methanol may present with symptoms ranging from being asymptomatic with an elevated osmolar gap to experiencing severe illness with end-organ toxicity, high anion gap metabolic acidosis, and potentially elevated lactic acid levels. Urinalysis results are generally normal, with no crystals found in the sediment [4]. Advanced diagnostic techniques, such as gas chromatography, can be employed to quantify methanol and its metabolites in biological fluids. Arterial blood gas analysis may reveal metabolic acidosis, a hallmark of methanol poisoning in severe cases. The osmolar gap, which measures the difference between the measured and calculated serum osmolarity, can also be a valuable diagnostic tool [4]. To evaluate neurological complications, which are a hallmark of methanol poisoning, radiological imaging, particularly brain imaging, may be necessary. Multidisciplinary team collaboration in evaluations is essential to confirm the diagnosis and guide appropriate therapeutic interventions.

All toxicology patients suspected of self-harm should receive a complete toxicological screening, an electrocardiogram, and a basic metabolic panel. Additional tests to consider include complete blood count; transaminase, lipase, and amylase levels; pregnancy status; and serum or urine ketone, lactate, ethanol, and salicylate concentrations. White blood cell counts, hemoglobin, and hematocrit are typically normal, but the mean corpuscular volume may be higher, which could be a sign of more serious poisoning and widespread cellular swelling. Elevated serum amylase levels can also indicate acute pancreatitis. Salicylate toxicity must be eliminated, particularly in patients who have metabolic acidosis. Because ethanol prevents methanol from being metabolized, the concentration of ethanol is also required. Although gas chromatography is a confirmatory method for measuring toxic alcohol concentrations, it is not readily available in all healthcare facilities. These concentrations, reported in mg/dL, typically peak soon after absorption and decrease via zero-order kinetics. The time of ingestion is important because the toxic alcohol concentration may not reflect toxicity levels if metabolism has progressed, as the metabolites are primarily responsible for the toxic effects. Assessing the formate concentration for methanol can be related to clinical signs of end-organ damage or acidosis. While a diagnosis is typically required sooner, obtaining dangerous alcohol concentrations frequently necessitates sending a serum sample to an external institution, which could take hours or days for findings. A patient with normal acid–base status shortly after methanol ingestion should be observed for at least 12 h, with serial basic metabolic panels performed every 2 to 4 h to monitor for the onset of metabolic acidosis and an elevated anion gap. This observation period should begin only after confirming that the patient’s ethanol concentration is undetectable. The 12 h observation period is considered the standard of care, based more on clinical experience than specific data, as acidosis typically develops within this timeframe following ingestion [3]. Diagnosing methanol poisoning can be challenging, particularly when the patient’s history is unclear, necessitating a high degree of clinical suspicion. Prompt detection and timely therapeutic interventions, including the use of ADH inhibitors, are critical for assessing and potentially reversing the damage caused by formic acid.

## 6. Diagnostic Testing

Methanol levels are typically assessed via gas chromatography or radioimmunoassay methods [4]. Laboratory assessments for methanol poisoning should include arterial blood gas analysis alongside blood samples. Urinalysis with microscopy is essential for detecting crystalluria, which may indicate ethylene glycol poisoning, although the absence of crystals has no diagnostic significance. When encountering patients with unexplained metabolic acidosis, especially after diabetic ketoacidosis and renal failure are ruled out, calculating the anion and osmolal gaps (OGs) can provide diagnostic clues [4,21]. Methanol increases serum osmolality at toxicity-related doses. This effect can be evaluated by calculating the OG with the following common formula:


Osmolal Gap = [Measured Osmolality] − [Calculated Osmolality]

Calculated osmolality = [1.86 × sodium concentration (mmol)] + [glucose concentration (mg/dL)/18] + [BUN concentration (mg/dL)/2.8] + [1.25 × ethanol (mmol/L)]

The normal range for OG in acute patients is 5 ± 14 mOsm/kg H_2_O [23]. An OG above 10 indicates the presence of exogenous osmoles. However, a cutoff value of 25 mOsm/kg H_2_O is considered effective [23,24]. In the initial stages or with ethanol coingestion, only OG may be elevated, as methanol has not yet been metabolized into formate. In the later stages, when methanol is converted to formate, the anion gap increases, whereas the OG may normalize. Detecting formate might then be necessary for confirmation. Additionally, brain scans such as CT or MRI might reveal late-stage findings such as necrosis in the putamenal areas due to methanol poisoning [4].

## 7. Treatment

**Figure 3:**
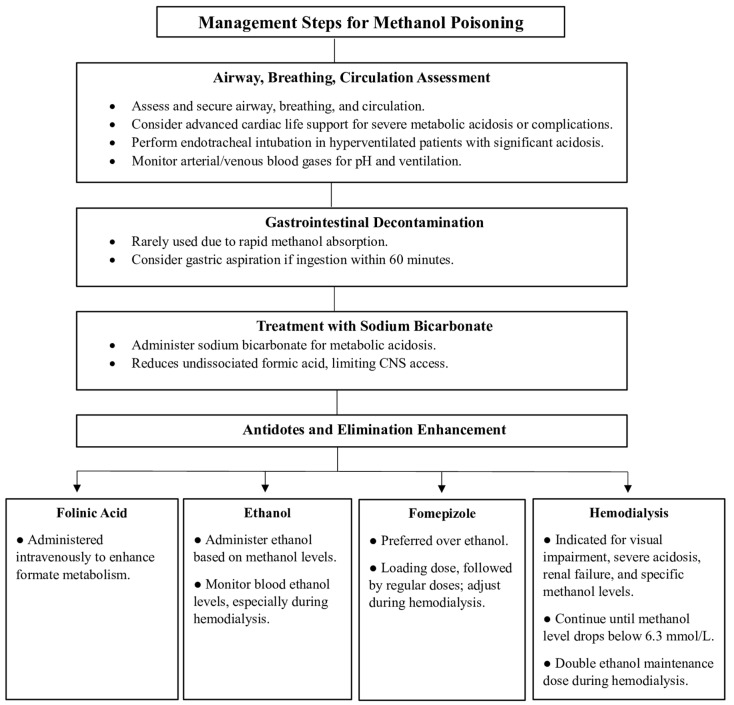
The clinical management of methanol poisoning.

Methanol poisoning is a medical emergency that can lead to severe complications such as permanent neurological damage, blindness, and death. Therefore, prompt recognition and intervention are needed. Treatment decisions are based only upon clinical suspicion and readily available laboratory data (Figure 3).

### 7.1. Airways, Breathing, and Circulation

The first step in managing methanol toxicity is to secure the patient’s airway, breathing, and circulation. Advanced cardiac life support measures are considered in cases of severe metabolic acidosis, complications that threaten cardiac and respiratory functions, or standard treatments for methanol toxicity that have proven ineffective in stabilizing patients’ condition [6,25]. In hyperventilated patients with existing or suspected significant metabolic acidosis, endotracheal intubation is needed. In addition, it is crucial to monitor arterial/venous blood gases closely to evaluate pH and ensure adequate ventilation and oxygenation [7].

### 7.2. Gastrointestinal Decontamination

Gastrointestinal decontamination is not commonly used when treating methanol poisoning, as methanol is rapidly absorbed. However, gastric aspiration via flexible nasogastric tubing can be an option if a patient ingests a large amount of methanol within 60 min of admission. Other methods, such as activated charcoal, gastric lavage, and syrup with ipecac, do not have a role in alcohol toxicity [8].

### 7.3. Treatment with Sodium Bicarbonate

The immediate and aggressive treatment of methanol poisoning involves the administration of sodium bicarbonate with the aim of completely correcting acidosis. Notably, bicarbonate treatment lowers the quantity of undissociated formic acid to diminish the access of formate to the CNS and thereby reduce toxicity [25]. It also treats metabolic acidosis. Initial infusion may require as many as 400 to 600 milliequivalents (mEq) within the first few hours. The final goal of treatment is to maintain the arterial/venous pH above 7.35 when infusion is discontinued.

### 7.4. Antidotes and Elimination Enhancement

The inhibition of ADH prevents the metabolism of methanol into its more toxic metabolites. The ADH inhibitors used for managing methanol toxicity are fomepizole and ethanol. They are indicated if the serum methanol level is >20 mg/dL, there is a recent history of ingesting toxic amounts of methanol and a serum osmol gap > 10, or there is strong clinical suspicion of methanol toxicity with at least two of the following criteria: arterial pH < 7.3, serum bicarbonate < 20 meq/L (mmol/L), an osmol gap > 10, and the presence of urinary oxalate crystals. If obtaining a methanol level is challenging and interpreting anions and OG is difficult, initiating ethanol or fomepizole therapy is recommended for any patient with metabolic acidosis and symptoms or a history of potential toxic alcohol ingestion [1,6,26].

#### 7.4.1. Folinic Acid

Increasing additional formate metabolism may be facilitated by intravenous folinic acid administration at doses ranging from 1 mg/kg to 50 mg every 4 h. If folinic acid is not available, folic acid at the same dosage can serve as an alternative [4].

#### 7.4.2. Ethanol

A therapeutic blood ethanol level of about 22 mmol/L (100 mg/dL) is recommended. However, because there is dynamic competition with the liver’s ADH enzyme, the amount of ethanol needed to block methanol metabolism is dependent on the level of methanol present at the time. The molar ethanol concentration should be at least one-fourth of the molar methanol concentration if the blood methanol level is known [1,4,25,26]. By giving a bolus dose of 600–800 mg/kg of 10% ethanol in D5W, followed by a maintenance dose of 66–154 mg/kg/h as an intravenous infusion of the 10% ethanol in D5W, or by taking 20% ethanol diluted in orange juice orally, for instance, a blood ethanol level of 100 mg/dL can be achieved. Monitoring blood ethanol levels is crucial, especially during hemodialysis. If ethanol was coingested with methanol and the blood ethanol level initially was >22 mmol/L (100 mg/dL), the bolus dose of ethanol can be skipped. Typically, the maintenance dose of ethanol should be doubled during hemodialysis to prevent the resumption of methanol metabolism when ethanol levels decrease, leading to worsening toxicity despite hemodialysis [1,25,26,27]. Ethanol therapy should continue until the diagnosis of toxic alcohol ingestion is ruled out or specific criteria related to blood pH and the serum alcohol concentration are met. In addition, owing to associated risks, patients should be treated in a critical care setting, ideally with a central venous catheter and an infusion pump. In the absence of fomepizole and pharmaceutical-grade IV ethanol, oral ethanol administration can be considered with appropriate dilutions, but it may lead to side effects such as gastritis and vomiting. Combining ethanol with fomepizole therapy offers no added benefit in methanol and ethylene glycol poisoning cases [28].

#### 7.4.3. Fomepizole

Fomepizole is a commercially available treatment for methanol poisoning and is preferred over ethanol. A loading dose of fomepizole (15 mg/kg IV) followed by four maintenance doses 12 h apart (10 mg/kg IV) and 15 mg/kg every 12 h thereafter is the recommended dosing regimen for fomepizole. If hemodialysis is initiated, the fomepizole dosing frequency should be increased every 4 h [29,30,31]. Fomepizole therapy should continue until the diagnosis of toxic alcohol ingestion is ruled out or a controlled blood pH and decreased serum alcohol concentration to <20 mg/dL is achieved.

Fomepizole, due to its higher affinity for ADH compared to ethanol, offers several clinical advantages. These include a broader therapeutic index, longer duration of action, simplified dosing, and more consistent pharmacokinetics. Additionally, fomepizole is associated with fewer side effects compared to ethanol [8]. However, its primary drawback is its high cost. Despite this, significant cost savings can be achieved by reducing the need for hemodialysis, ethanol infusions, prolonged hospital stays, and intensive care unit admissions [15].

#### 7.4.4. Hemodialysis

Hemodialysis is a highly effective method for eliminating methanol and formate while correcting metabolic acidosis. The primary indication for hemodialysis is any degree of visual impairment in a patient with metabolic acidosis or detectable methanol levels. Other indications include severe metabolic acidosis (especially if unresponsive to previous therapies), deteriorating vital signs and electrolyte imbalances despite optimal treatment, renal failure, a blood methanol level exceeding 15.6 mmol/L (50 mg/dL), and ingestion of more than 1 g/kg methanol. In severe poisonings characterized by marked metabolic acidosis (base deficit > 20 mmol/L) and visual disturbances, hemodialysis is strongly recommended to rapidly remove methanol and formate [4,27].

Hemodialysis is typically continued until the blood methanol level falls below 6.3 mmol/L (20 mg/dL) and metabolic acidosis resolves. In cases where methanol level testing is unavailable, hemodialysis should proceed for at least 8 h or until the osmolar gap normalizes in two consecutive samples taken 1 h apart (after accounting for any osmolal contribution from ethanol) [1,26,27]. This method has proven effective in optimizing dialysis time, particularly during large outbreaks or when dialysis resources are limited. Serum formate concentrations may also serve as an alternative guide for determining the need for hemodialysis. Additionally, doubling the maintenance dose of ethanol during hemodialysis is generally recommended, as failing to do so may allow methanol metabolism to resume when ethanol levels drop, potentially worsening toxicity despite ongoing hemodialysis [27].

## 8. Conclusions

Methanol (CH_3_OH) is a widely used industrial and household alcohol that poses significant health risks upon exposure. Despite its extensive use, methanol poisoning remains a critical public health concern globally, often resulting from accidental or intentional ingestion and outbreaks linked to contaminated beverages. Methanol toxicity stems from its metabolic conversion to formaldehyde and formic acid, leading to severe metabolic acidosis and multiorgan damage, including profound CNS effects and visual impairments.

Epidemiological data underscore the widespread impact of methanol poisoning, with alarming case fatality rates reported in various countries. Comprehensive prevention and effective management strategies are urgently needed to address the significant morbidity and mortality associated with methanol poisoning.

The clinical manifestations of methanol toxicity vary between adult and pediatric populations and between acute and chronic exposure. Adults typically present with gastrointestinal and neurological symptoms, whereas pediatric patients often exhibit more severe outcomes due to differences in metabolism and body weight. Acute poisoning generally involves a large, single ingestion, leading to rapid symptom onset, whereas chronic poisoning results from small, repeated exposures, causing insidious, progressive symptoms.

The diagnosis of methanol poisoning involves a combination of clinical evaluation, laboratory testing, and advanced diagnostic techniques. The identification of metabolic acidosis, elevated anion and OG levels, and confirmation through methanol and formate levels are critical for accurate diagnosis. Timely intervention is crucial, and the management of methanol poisoning includes securing the airway, breathing, and circulation; addressing metabolic acidosis with sodium bicarbonate; and administering antidotes, such as fomepizole or ethanol. Hemodialysis plays a pivotal role in eliminating methanol and its toxic metabolites, especially in severe cases with significant metabolic acidosis or visual disturbances.

In conclusion, the complexity of methanol poisoning necessitates a comprehensive approach encompassing early recognition, prompt intervention, and coordinated care among healthcare providers. Increased awareness, effective prevention strategies, and timely treatment protocols are essential to mitigate severe health consequences and improve patient survival and recovery.
